# Food Allergen Carryover Within the Edible Insect Supply Chain: A Pilot Plant Investigation

**DOI:** 10.3390/foods15091528

**Published:** 2026-04-28

**Authors:** Clara Tramuta, Carla Ferraris, Samantha Lupi, Alessandra Provera, Irene Floris, Sara Morello, Aitor Garcia-Vozmediano, Cristiana Maurella, Daniela Manila Bianchi

**Affiliations:** 1 Istituto Zooprofilattico Sperimentale del Piemonte Liguria e Valle d’Aosta, 10154 Turin, Italy; clara.tramuta@izsplv.it (C.T.); samantha.lupi@izsplv.it (S.L.); alessandra.provera@izsplv.it (A.P.); irene.floris@izsplv.it (I.F.); sara.morello@izsplv.it (S.M.); aitor.garciavozmediano@izsplv.it (A.G.-V.); cristiana.maurella@izsplv.it (C.M.); manila.bianchi@izsplv.it (D.M.B.); 2 Centro di Referenza Nazionale per la Rilevazione Negli Alimenti di Sostanze e Prodotti che Provocano Allergie e Intolleranze (CreNaRiA), 10154 Turin, Italy

**Keywords:** edible insects, ELISA, food allergens, novel food, real-time PCR

## Abstract

The aim of this study was to assess whether edible insects reared on substrates containing food allergens can carry these allergens into the final product, and to evaluate the effectiveness of a pre-harvest fasting period in reducing this risk to provide consumer protection. *Hermetia illucens* larvae, chosen as the model species, were grown on substrates containing 10% of each of the following food allergens: peanut, almond, soy, celery, and gluten. At the end of the feeding period, larvae were sampled at T0 (end of feeding), T1 (24 h fasting), T2 (48 h fasting) and tested by real-time PCR and ELISA to detect allergen residues. Positive results were observed by real-time PCR for soy (mean Ct: 28.84 at T0, 29.4 at T1, 30.95 at T2), celery (mean Ct: 26.74 at T0, 26.90 at T1, 29.77 at T2) and almond (Ct 33.96 at T0 and mean Ct: 34.01 at T1). Soy presence was also confirmed by ELISA test. Insects may represent an alternative food source; however, their use requires careful evaluation due to the potential presence of allergens. Our results showed that insects may contain allergens originating from their feeding substrates, potentially triggering a response in allergic consumers.

## 1. Introduction

As the global population projected to reach 9 billion by 2037, the resulting surge in food demand presents significant global challenges [1]. Consequently, there is growing interest—particularly within the European Union—in alternative protein sources, such as edible insects. Insects offer substantial nutritional, environmental, and economic advantages, as they stem from a livestock sector characterized by lower energy requirements and reduced waste production [2].

Regulation (EU) 2015/2283 [3] defines and governs the authorization process for “novel foods”. To date, four insect species are authorized for human consumption in Europe: the house cricket (*Acheta domesticus*), the lesser mealworm (*Alphitobius diaperinus*), the migratory locust (*Locusta migratoria*), and the yellow mealworm (*Tenebrio molitor*). These species are prized for their high protein content, balanced amino acid profiles, and efficient feed conversion [4,5]. Other species, such as the Black Soldier Fly larvae (*Hermetia illucens*), are currently under evaluation as this Regulation is subject to ongoing review [6]. While some emerging species are currently categorized under EU law primarily for feed and industrial applications (Regulation (EU) 2017/893 [7]), they represent a strategic focus for scientific research due to their robust metabolic profiles and bioconversion efficiency. Rigorous characterization of these “bioconverters” provides essential baseline data on nutritional and safety profiles, a fundamental prerequisite for future inclusion in the list of insects authorized for human consumption [8].

The integration of insects into the Western diet is strictly contingent upon guaranteed safety standards. In this context, the growth substrate is the primary determinant of the chemical and biological integrity of the resulting food products. Studies show that harmful substances can migrate from feed into insects through (i) chemical contaminants (e.g., dioxins and polycyclic hydrocarbons), (ii) the bioaccumulation of heavy metals in tissues, and (iii) the colonization of the insect by pathogenic bacteria from the substrate [9,10].

Regarding the allergic safety of these products, cross-reactivity with other arthropods is well-documented; specifically, insects contain allergens like tropomyosin and arginine kinase, which cause cross-sensitization to crustaceans and dust mites as these allergenic proteins exhibit IgE-binding cross-reactivity with homologous allergens found in such organism [11,12]. This poses a potential health risk to sensitive individuals, making clear safety protocols and labelling essential [13]. A critical concern involves the transfer of food allergens, such as gluten and milk proteins, from the substrate to the insects [8,13]. Consequently, assessing the probability of allergen carry-over is essential for accurate and transparent food labeling. To minimize the presence of undesirable substances, pre-harvest fasting has proven highly effective. A recent study demonstrated that a 48-h fasting period significantly reduces gluten content in *Tenebrio molitor* larvae to below the 20 ppm “gluten-free” threshold [13]. This issue is particularly pressing given that common rearing substrates often rely on major allergens. Substrates may incorporate wheat, milk, soy and peanut-derived by-products, or even crustacean processing waste in certain experimental or Extra-EU scenarios [8,14]. Consequently, the presence of even trace amounts represents a risk for sensitive consumers.

Despite the knowledge that the substrate influences safety, data on the carry-over of specific allergens (such as soy, peanuts, celery or almonds) are still scarce or inconsistent in the current literature. Consequently, assessing the probability of allergen carry-over is essential for accurate food labeling. Therefore, the aim of this pilot study was to evaluate the role of insect-based novel foods as vehicles for plant-derived food allergens through experimental trials on the species *Hermetia illucens* reared on growth substrates containing specific allergenic ingredients. Therefore, the effectiveness of a pre-harvest fasting period in reducing allergenic risk for consumer protection was also evaluated.

## 2. Materials and Methods

### 2.1. Substrate Administration

The initial phase of the study was conducted at a pilot plant located in Piedmont (Northwest Italy), which rears insects of the species *Hermetia illucens,* a commonly used insect species in large-scale [15]. The larvae were reared for 15 days on five growth substrates consisting of rice and maize, each containing 10% (*w*/*w*) of one of the following food allergens: peanut, almond, soy, celery and gluten. This concentration was selected to simulate a high exposure ‘worst-case scenario’, ensuring that potential protein carry-over remained within the detectable range of analytical tools and allowing for a robust evaluation of the fasting period’s effectiveness.

### 2.2. Sample Preparation

To evaluate the potential persistence of traces of the allergens in the insects’ intestinal tract, once the substrate administration period ended, larvae were subjected to a fasting period. Three different sampling intervals were collected: T0 (end of feeding, in triplicate), T1 (24 h fasting, in triplicate), and T2 (48 h fasting, in triplicate). Subsequently, the samples were frozen (−20 °C), dried (80 °C for 2 h and 65 °C for 17 h), and ground. These temperatures were chosen to simulate standard industrial drying practices. For each of the three time points, a negative control group consisting of larvae reared on allergen-free substrates was also tested.

### 2.3. Laboratory Analysis (Real-Time PCR and ELISA Test)

Samples of larvae reared on substrates with and without allergens were analyzed to assess the presence of the five food allergens using: molecular biology techniques (Real-Time PCR) to detect peanut, almond, soy, and celery DNA, and enzyme-linked immunosorbent assays (ELISA) for the quantitative detection of gluten protein and the qualitative detection of soy protein. The techniques used have been selected for their accreditation and regular application for the specific analytical tasks at our laboratory.

#### 2.3.1. DNA Extraction and Real-Time PCR

DNA was extracted using the commercial ION Force DNA Extractor Fast kit (Generon, San Prospero, MO, Italy). Briefly, 5 g of sample were mixed with 20 mL of solution A and incubated for 1 h at 85 °C in a water bath or thermomixer. Following centrifugation at 10,000 rpm for 10 min, the aqueous supernatant was transferred to a 50-mL bottle and mixed with an equal volume of buffer E. After a second centrifugation (10,000 rpm for 10 min), the subnatants were treated with 7.5 mL of buffer T and 20 µL of glacial acetic acid. Finally, the columns were assembled on a vacuum chamber for purification, followed by two washes with 1 mL of buffer P. To remove residual ethanol, the columns were centrifuged at 7000 rpm for 5 min. Finally, DNA was eluted in 100 µL of buffer D via two-step centrifugation: 30 s at 500 rpm and 5 min at 14,000 rpm. Real-time PCR assays for peanut, celery, soy, and almond targets were prepared in a final volume of 20 µL, containing 15 µL of SPECIALfinder MC Working Master Mix (Generon, San Prospero, MO, Italy) and 5 µL of DNA. Amplification was performed by selecting the target (FAM) and internal amplification control (IAC) detectors. Real-time PCR was performed on a CFX96 system (Bio-Rad, Richmond, CA, USA) using the following conditions: initial denaturation at 95 °C for 3 min, 35 cycles of denaturation at 95 °C for 5 s, annealing at 60 °C for 10 s, and extension at 72 °C for 20 s.

#### 2.3.2. ELISA Test

The commercial RIDASCREEN^®^ Gliadin ELISA kit (R-Biopharm, Darmstadt, Germany) was used for gluten detection, with a LOD of 5 mg/kg (ppm). Sample preparation and analysis were conducted in accordance with the manufacturers’ instructions. Briefly, 0.25 g of sample was extracted with 2.5 mL of extraction buffer at 50 °C for 40 min, followed by the addition of 7.5 mL of 80% ethanol, 1 h incubation at room temperature, and centrifugation at 2500× *g* for 10 min. The supernatant was diluted 1:500, transferred to microplate wells with standards and controls, and incubated with conjugated antibody, with washes in between. Then, substrate and chromogen were added and incubated in the dark; absorbance was measured at 450 nm. For the detection of soy, the commercial RIDASCREEN^®^ FAST Soya kit (R-Biopharm, Darmstadt, Germany) was used instead. Also, in this case, sample preparation and analysis were performed according to the manufacturers’ instructions. Proteins were extracted from 1 g of sample using 2.5 mL of Extractor 3 and 17.5 mL of allergen extraction buffer, followed by incubation at 100 °C for 10 min and centrifugation. The supernatant was diluted 1:5, and 100 μL of extracts, standards and controls were added to microplate wells, sequentially incubated with enzyme-conjugated antibody and substrate–chromogen. The reaction was stopped with 100 μL of stop solution, and absorbance was measured at 450 nm.

### 2.4. Statistical Analyses

Due to the pilot nature of the study and the limited number of independent replicates (*n* = 3 per time point), all analytical results were evaluated using a categorical approach. Real-time PCR and ELISA outcomes were classified as positive or negative (above or below the limit of detection), and analyses were performed on binary data.

The primary objective was to assess whether larvae reared on allergen-containing substrates (Treatment group) showed a higher frequency of allergen detection compared with larvae reared on allergen-free substrates (Control group). For this purpose, analyses were conducted separately for each allergen. To increase the informativeness of the comparison in this small dataset, observations from all sampling times (T0, T1, T2) were pooled within each allergen, and detection frequencies between Treatment and Control groups were compared using Fisher’s exact test.

As a secondary exploratory analysis, the effect of fasting time (T0, T1, T2) on allergen detection was evaluated within the Treatment group for each allergen. Global comparisons across time points were performed using Fisher’s exact test for contingency tables, and pairwise comparisons between time points were explored when applicable. For pairwise comparisons across time points, *p*-values were adjusted for multiple testing within each allergen using the Bonferroni correction method. Adjusted *p*-values were interpreted alongside unadjusted results.

In cases where no variability in outcome was observed (i.e., all samples were either positive or negative), statistical comparisons were considered non-informative and were not interpreted. All statistical analyses were performed using R software (version 4.5.3). Statistical significance was set at *p* < 0.05.

## 3. Results

### 3.1. Real-Time PCR

**Figure 1:**
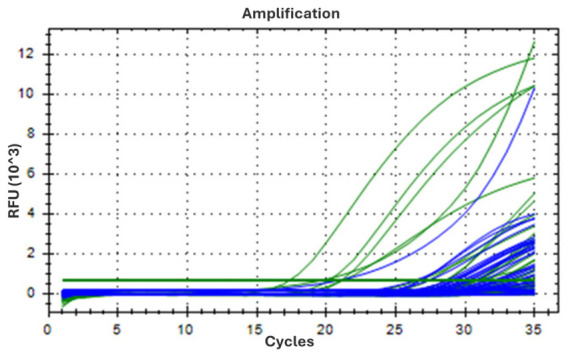
Real-time PCR amplification curves for the detection of food allergens in the intestinal tract of insect larvae.

Real-time PCR analysis revealed distinct patterns of allergen detection depending on the substrate. Soy DNA was consistently detected in all Treatment samples across the three experimental time points (T0–T2), with comparable Ct values (mean Ct: 28.84 at T0, 29.4 at T1, 30.95 at T2), whereas all control samples were negative (Table 1). A similar trend, although less pronounced, was observed for celery, which was detected in 7 out of 9 Treatment replicates overall, with Ct values indicating comparable amplification across time points (26.74 at T0, 26.90 at T1 and 29.77 at T2; Table 1). When results were analyzed as binary outcomes, these patterns translated into a significantly higher detection frequency in the Treatment group compared with controls for both soy (9/9 vs. 0/9, *p* < 0.001) and celery (7/9 vs 0/9, *p* = 0.002), supporting the occurrence of allergen carryover from the rearing substrate (Figure 1).

In contrast, almond DNA was detected in one replicate at T0 (Ct: 33.96) and in two replicates at T1 (mean Ct: 34.01), although this differences did not reach statistical significance (*p* = 0.206). Peanut DNA was not detected in any sample from either group (Table 1). As expected, the negative controls (C1, C2, and C3) sampled at T0, T1, and T2 showed no fluorescence signals for the sought targets (Figure 1, Table 1).

Despite slight variations in detection frequencies and Ct values across time points for some allergens, particularly almond and celery, exploratory analyses within the Treatment group did not show statistically significant differences in allergen detection between T0, T1 and T2 (*p* > 0.05).

### 3.2. ELISA Test

ELISA analysis showed a clear dichotomy between the investigated allergens. Gluten was consistently below the LOD (<5 ppm) in all samples, both in Treatment and Control groups, at all time points (Table 2). On the contrary, regarding the detection of soy proteins, results above the LOD (>2.5 ppm) were obtained in all three replicates at T0, T1 and T2, while all control samples remained negative. This consistent pattern resulted in a statistically significant difference in detection frequency between groups (9/9 vs. 0/9, *p* < 0.001), confirming the presence of soy at the protein level and corroborating the PCR findings.

No variation in ELISA outcomes was observed across time points within the Treatment group for either allergen; therefore, time-based comparisons were not informative.

## 4. Discussion

This pilot study evaluated the potential carryover of food allergens from rearing substrates to *H. illucens* larvae and the effect of a pre-harvest fasting period on allergen persistence. Overall, our findings indicate that allergen transfer can occur, although its extent appears to be strongly dependent on the specific allergen. In particular, consistent detection of carryover was observed for soy and, to a lesser extent, celery, whereas almond showed only sporadic detection and peanut was not detected in any larval sample. Moreover, no clear variation in allergen detection was observed across time points, suggesting that a reduction in detectability during the 48-h fasting period could not be demonstrated within the limits of this study.

While insects represent a promising alternative food source, their integration into the food chain requires a rigorous evaluation of potential allergens. Furthermore, since insects are typically consumed whole, including the intestinal tract, a stringent safety assessment regarding food allergens derived from the rearing substrate is crucial. The study may suggest that mitigation strategies such as fasting can reduce, but not necessarily eliminate, this risk of allergen carryover. These findings align with Frigerio et al. (2020) [16], who observed that while certain proteins are degraded during the larval digestive process, DNA fragments and stable protein fractions from the substrate can persist, especially in the absence of an adequate starvation period.

The national reference center for the detection of food allergens and substances causing food intolerance (CReNaRiA, Centro di Referenza Nazionale per la Rilevazione negli Alimenti di Sostanze e Prodotti che provocano Allergie e Intolleranze) was designed to address the growing demand for consumer protection within the Italian and European legislative frameworks. The Center is responsible for the development, validation, and accreditation of innovative analytical methods, prioritizing technological advancement in food allergen research. Among the recent investigations undertaken by CReNaRiA, two key areas stand out: (i) the validation of analytical techniques for detecting emerging allergens; (ii) evaluating the role of edible insects both as potential allergens and as carriers of food allergens. In line with these objectives, recent investigations into novel foods have yielded significant insights. The prevailing analytical strategies for detecting food allergens are primarily based on ELISA and PCR approaches [17,18,19,20,21,22]. However, the effectiveness of these methods depends heavily on the nature of the sample. Regarding the ELISA test, certain food matrices can cause interference or cross-reactivity, compromising the accuracy of the test. Furthermore, the reliability of ELISA decreases drastically in products subjected to thermal treatment; cooking denatures or fragments the target proteins, preventing specific antibodies from recognizing and detecting them correctly [23,24]. Conversely, PCR-based methods often perform better in processed or cooked products. This is because DNA possesses superior thermal stability compared to proteins, remaining detectable even after intense heating processes [23,25]. In the present study, the concordance between PCR and ELISA results for soy strengthens the evidence of allergen persistence, while the absence of detectable gluten by ELISA should be interpreted cautiously, as concentrations may be below the method’s limit of detection (5 ppm) rather than completely absent.

A key strength of this work lies in the evaluation of multiple allergens, an aspect of relevance given the current paucity of data in this field and the urgent need for robust evidence to ensure consumer safety. Our findings hint that when insects are reared on substrates containing allergens, these may be transferred to the larvae and potentially to derived food products, although the extent of this process varies among allergens. The persistence of soy and celery across sampling points is consistent with a relatively higher stability or resistance to degradation under the conditions tested. In contrast, almond was detected only at low frequency and limited to earlier time points, suggesting reduced persistence or lower detectability.

Interestingly, a discrepancy was observed between the substrate and the final product for peanut, which was not detected in larval samples despite its presence in the feed. This could be attributed to the physicochemical nature of this allergen; being lipid-rich and highly sensitive to thermal processing, it may undergo significant denaturation during the drying and milling phases of larval processing. According to a recent study [26], peanut allergens (such as Ara h 2, Ara h 3 and Ara h 6) can resist digestion in the intestinal tract, highlighting the complex interplay between allergen structure, matrix and processing conditions in determining their detectability and immunogenic potential. In addition, previous studies have shown that heat treatment can considerably affect the detectability of peanut components, particularly at the level of DNA extraction [27], supporting that matrix effects and processing play a critical role. Therefore, the specific contribution of fasting to this observation cannot be isolated based on the present data.

With regard to fasting, the absence of clear differences in allergen detection across time points suggests that its effect may be limited or variable depending on the allergen. While fasting is commonly proposed as a mitigation strategy to reduce intestinal content, the persistence of certain allergens indicates that residues may not be confined to the gut lumen and may instead be associated with larval tissues or exhibit slower degradation kinetics. These observations highlight that the effectiveness of fasting is likely allergen-specific and influenced by multiple factors, including substrate composition and processing conditions.

These results have direct implications for Food Business Operators involved in insect rearing. To guarantee consumer safety and ensure transparent labeling, two primary strategies emerge: (i) the use of naturally allergen-free substrates (e.g., maize or rice-based by-products); and (ii) the implementation of appropriate precautionary allergen labeling when allergen-containing substrates are used. More broadly, the concept of “allergen-free” insect products cannot be generalized and should be evaluated on a case-by-case basis, taking into account substrate composition, analytical sensitivity and processing conditions. Although limited by the small sample size and the exploratory nature of the study, these findings provide a useful basis for future research aimed at better understanding allergen persistence and defining effective risk mitigation strategies.

## 5. Conclusions

With this pilot study it was possible to show that insects may contain allergens originating from their feeding substrates, potentially triggering a response in allergic consumers. The persistence of certain food allergens after 48 h of fasting (soy, almond and celery) may be contingent upon the specific allergen in question. Although the edible insect sector is not yet fully established in Italy, we believe it is important to continue this type of investigation as these products are already on the market and their consumption could increase significantly in the coming years. The limitations of the study are acknowledged, including the relatively modest sample size and the preliminary nature of the investigation; nevertheless, the findings provide a valuable foundation for future research on the allergen-related safety of edible insects. It will be worthwhile investigating in future studies whether the allergens originate from intestinal contents or from insect tissues.

## Figures and Tables

**Table 1:** Results of real-time PCR analyses for the detection of food allergens in insect larvae.

Samples (Replicates)		Real-Time PCR (Ct Cycle)			
		PEANUT	ALMOND	SOY	CELERY
C1 T0	Control	negative (>35)	negative (>35)	negative (>35)	negative (>35)
C2 T0		negative (>35)	negative (>35)	negative (>35)	negative (>35)
C3 T0		negative (>35)	negative (>35)	negative (>35)	negative (>35)
1 T0	10% target	negative (>35)	positive (33.96)	positive (27.63)	positive (27.26)
2 T0		negative (>35)	negative (>35)	positive (30.07)	negative (>35)
3 T0		negative (>35)	negative (>35)	positive (28.83)	positive (26.23)
C1 T1	Control	negative (>35)	negative (>35)	negative (>35)	negative (>35)
C2 T1		negative (>35)	negative (>35)	negative (>35)	negative (>35)
C3 T1		negative (>35)	negative (>35)	negative (>35)	negative (>35)
1 T1	10% target	negative (>35)	positive (34.61)	positive (30.85)	negative (>35)
2 T1		negative (>35)	positive (33.41)	positive (28.49)	positive (27.28)
3 T1		negative (>35)	negative (>35)	positive (28.86)	positive (26.53)
C1 T2	Control	negative (>35)	negative (>35)	negative (>35)	negative (>35)
C2 T2		negative (>35)	negative (>35)	negative (>35)	negative (>35)
C3 T2		negative (>35)	negative (>35)	negative (>35)	negative (>35)
1 T2	10% target	negative (>35)	negative (>35)	positive (28.72)	positive (31.44)
2 T2		negative (>35)	negative (>35)	positive (31.98)	positive (27.28)
3 T2		negative (>35)	negative (>35)	positive (32.15)	positive (30.58)

T0: end of feeding; T1: post-24 h fasting; T2: post-48 h fasting.

**Table 2:** Results of ELISA test for the detection of food allergens in insect larvae.

Samples (Replicates)		ELISA Test (ppm)	
		GLUTEN	SOY
C1 T0	Control	negative (<5)	negative (<2.5)
C2 T0		negative (<5)	negative (<2.5)
C3 T0		negative (<5)	negative (<2.5)
1 T0	10% target	negative (<5)	positive (>2.5)
2 T0		negative (<5)	positive (>2.5)
3 T0		negative (<5)	positive (>2.5)
C1 T1	Control	negative (<5)	negative (<2.5)
C2 T1		negative (<5)	negative (<2.5)
C3 T1		negative (<5)	negative (<2.5)
1 T1	10% target	negative (<5)	positive (>2.5)
2 T1		negative (<5)	positive (>2.5)
3 T1		negative (<5)	positive (>2.5)
C1 T2	Control	negative (<5)	negative (<2.5)
C2 T2		negative (<5)	negative (<2.5)
C3 T2		negative (<5)	negative (<2.5)
1 T2	10% target	negative (<5)	positive (>2.5)
2 T2		negative (<5)	positive (>2.5)
3 T2		negative (<5)	positive (>2.5)

T0: end of feeding; T1: post-24 h fasting; T2: post-48 h fasting.

## Data Availability

The original contributions presented in this study are included in the article. Further inquiries can be directed to the corresponding author.
